# Low Profile Monopole Meander Line Antenna for WLAN Applications

**DOI:** 10.3390/s22166180

**Published:** 2022-08-18

**Authors:** Husam Hamid Ibrahim, Mandeep Jit Singh, Samir Salem Al-Bawri, Sura Khalil Ibrahim, Mohammad Tariqul Islam, Mohamed S. Soliman, Md Shabiul Islam

**Affiliations:** 1 Department of Electrical, Electronic and Systems Engineering, Faculty of Engineering and Built Environment, Universiti Kebangsaan Malaysia (UKM), Bangi 43600, Selangor, Malaysia; 2 Space Science Centre, Institute of Climate Change, Universiti Kebangsaan Malaysia (UKM), Bangi 43600, Selangor, Malaysia; 3 Department of Electronics & Communication Engineering, Faculty of Engineering & Petroleum, Hadhramout University, Al-Mukalla 50512, Yemen; 4 Department of Electrical Engineering, College of Engineering, Taif University, P.O. Box 11099, Taif 21944, Saudi Arabia; 5 Department of Electrical Engineering, Faculty of Energy Engineering, Aswan University, Aswan 81528, Egypt; 6 Faculty of Engineering, Multimedia University, Persiaran Multimedia, Cyberjaya 63100, Selangor, Malaysia

**Keywords:** monopole antenna, meander line, WLAN, reflection coefficient

## Abstract

An antenna assumes a significant role in expanding the levels of communication to meet the demands of contemporary technologically based industry and private data services. In this paper, a printed compact meander line patch antenna array for wireless local-area network (WLAN) applications in the frequency span of 2.3685–2.4643 GHz is presented. The impedance matching of the antenna is generated by applying a partial rectangular-shaped ground plane backside of the meander line antenna. The proposed antenna evolved on the Rogers RT5880 substrate with a dielectric permittivity of 2.2, and the height of the substrate was 1.575 mm to accomplish the lowest possible return loss. The proposed antenna was developed to achieve particular outcomes, for example, voltage standing wave ratio (VSWR) 1.32, reflection coefficient 20 dB with a bandwidth of 94.2 MHz, a gain of 2.8 dBi, and an efficacy measurement of 97%. This antenna is appropriate for WLAN applications that utilize a 2.4 GHz resonance frequency. The overall dimensions of the antenna are 15 mm × 90.86 mm.

## 1. Introduction

Modern wireless communication systems are rapidly evolving, necessitating the development of innovative, multiband, and compact antennas. Since the last decade, mobile devices such as handheld computers and intelligent phones have been using wireless local-area network (WLAN) and worldwide interoperability for microwave access (WiMAX) technologies [1]. The antenna ought to have a high gain in order to receive available energy, and it must also be efficient in order to reduce losses [2]. The antenna is used in a variety of wireless electronic devices of various sizes [3]. Due to the limited space available for antennas in modern communication devices, antenna designers strive for compact antenna designs that meet wireless standards’ preferred bandwidths. Designing a very compact antenna with good radiation performance is difficult due to limited space for multi-standard integration and the requirement for antenna geometry modification [4]. There are several techniques for reducing antenna size, including the use of high primitive substrates, shorting pins, and meander line antenna designs. The Meander line is the best solution for wireless communication applications such as radio frequency identification tags, USB Dongles, Bluetooth headsets, mobile phones, and many more [5]. In a variety of applications, meander-line antennas have been investigated for miniaturization [6]. Because it introduces to the propagation time of an electromagnetic wave from the feed point to the antenna’s end, this type of miniaturisation technique will result in even smaller antennas electrically [7]. Monopole antennas have a high radiation efficiency, a broad impedance bandwidth, a low profile, are inexpensive, and have a simple structure. WLANs for high-resolution radars, imaging systems, military communication, and cognitive radio can easily incorporate these antennas [8]. The literature reports a variety of techniques for reducing the size of the monopole antenna, including the meander line technique.

In ref. [9], a lower-frequency UHF meander line antenna was designed using a metamaterial with a near-zero metamaterial density. In order to evaluate the antenna’s performance, it was introduced. A monopole antenna with an S-shape stepped meander line with a defected ground structure that can operate at a super-wide bandwidth was demonstrated [10]. The author in [11] illustrated a planar, compact dual-band antenna on a semi-flexible substrate for 2.45/5.85 GHz wireless body area networks. A superstrate of a double negative metamaterial plane has been used to improve the proposed antenna bandwidth and gain [12]. The printed monopole antenna in ref. [13] is accompanied by a meander line that acts as a near-field resonant parasitic (NFRP) element, reducing the overall size of the antenna. An improved wideband and compact flexible meander-line antenna for wearable electromagnetic head imaging systems was described by the author in [14]. A meander monopole antenna array based on an electromagnetic band gap (EBG) structure was demonstrated for multi-input multi-output (MIMO) applications [15]. Based on a symmetric split ring resonator, a metamaterial with epsilon negative (ENG) properties has been demonstrated to enhance the gain and directionality of broadband monopole antenna [16]. The reflect array element of the antenna is a slotted square patch with meander delay lines on each side of the square patch, and it operates in the X-band [17]. For microcell and femtocell applications, a closed-form, two-step localization technique was used. The developed algorithm as presented in [18] depended on received signal strength (RSS)-based indoor localization estimations using two antennas at 2.4 GHz, whereas antennas using Y-slot [19] and metamaterial [20] techniques were used reduce the size and enhance overall performance. However, the author in ref. [21] presented the antenna’s primary radiating element as a unit cell of a single-negative metamaterial structure with a meander line and a defected ground structure (DGS).

Printed monopole antennas are extensively utilised in numerous wireless communication devices because of the appealing features they offer, such as low cost, lightweight, compact size, and simplicity of operation with other systems [22]. Several numbers of antennas have been reported. A modified meander shape microstrip patch antenna with dimensions of 40 × 10 × 1.6 mm^3^ has been introduced for Internet of Things (IoT) applications at 2.4 GHz in the ISM (Industrial, Scientific, and Medical) band [23]. A concept for a small, wideband printed monopole antenna for wireless applications around 2.45 GHz was proposed in ref. [24]. A quarter circle, a rectangular section, and a flipped ground plane with the same shape as the patch make up the antenna. The use of left-handed double negative metamaterial (DNG) in a microstrip patch antenna resonator at 2.4 GHz was investigated [25]. In ref. [26], an experimentally tested printed antenna with a compressed meandered shape with dual able to operate frequencies for 2.5/5 GHz applications is introduced. For 2.45 GHz ISM band on-body WBAN applications, the author [27] presented a low-profile portable fractal antenna made of vinyl polymer-based polytetrafluoroethylene (PTFE) material properly known as Rogers 5880. The conformal antenna was demonstrated as a novel metamaterial-inspired compact cylindrical conformal dual-band antenna with a shunt fractal inductor and bottom patch. The conformal antenna is made up of three meander line type unit cells of composite right-handed and left-handed metamaterials for modification and compactness [28]. The author of [29] proposed an electrically compressed antenna with three open-circuit series stubs as an impedance matching network. To match the impedance, it has two twin-spiral slot-line radiation surfaces and three open-circuited series stubs based on a CPW feed line. The author in ref. [30] illustrated how to build a compact wideband and dual-band antenna. Fork-shaped strips, a rectangular slot, and a defective ground plane enable the presented antenna to operate in both wideband and dual bands simultaneously time. The author in [31] introduced the compressed bowtie antenna through the use of the meander method for miniaturization reasons at 2.45 GHz for medical applications. Table 1 provides an overview of the different meander antennas that have been written about in the relevant research so that they can be compared to the design that has been suggested.

In this paper, a compact monopole antenna using the meander line method at 2.4 GHz is introduced for WLAN applications. The proposed antenna consists of meandering lines in a horizontal shape with different lengths designed. It is clear that the proposed antenna provides a significant increase in bandwidth, gain, as well as efficiency, whereas compact size has also been achieved compared with the reported antenna in the literature. A detailed antenna design is discussed in the article, along with both simulated and measured results. A reasonable agreement can be observed between the simulated and measured results.

The geometry of the proposed antenna will be explained in Section 2, followed by the working principle in Section 3. The simulated and measured results of the proposed antenna will be demonstrated in Section 4 prior to the concluding remarks.

**Table 1:** A comparison of the recent literature concerning the proposed design.

Ref.	Frequency (GHz)	Gain (dBi)	Radiation Efficiency (%)	Return Loss (S11) dB	Bandwidth (MHz)	Dimension (mm^3^)
[23]	2.4	1.34	79	−19.11	146	40 × 10 × 1.6
[24]	2.4	1.97	97	-	-	38 × 10.8 × 0.8
[25]	2.4	1.8	-	−52	0.135	16 × 32.5 × 1.6
[26]	2.5	1.2	-	−25	200	10 × 19 × 1.6
[27]	2.45	2.06	75	−19	11	39 × 39 × 0.508
[28]	2.45	1.26	70	−10	970	40 × 30 × 0.787
[29]	2.4	1.33	65.3	-	180	12.45 × 13.05 × 0.8
[30]	2.45	1.78	-	-	235	19 × 31 × 1.58
[31]	2.45	1.47	88	−17	-	29 × 13.7 × 1.5
Proposed work	2.43	2.8	97	−20	94.2	15 × 90.86 × 1.575

## 2. Antenna Geometry

**Figure 1:**
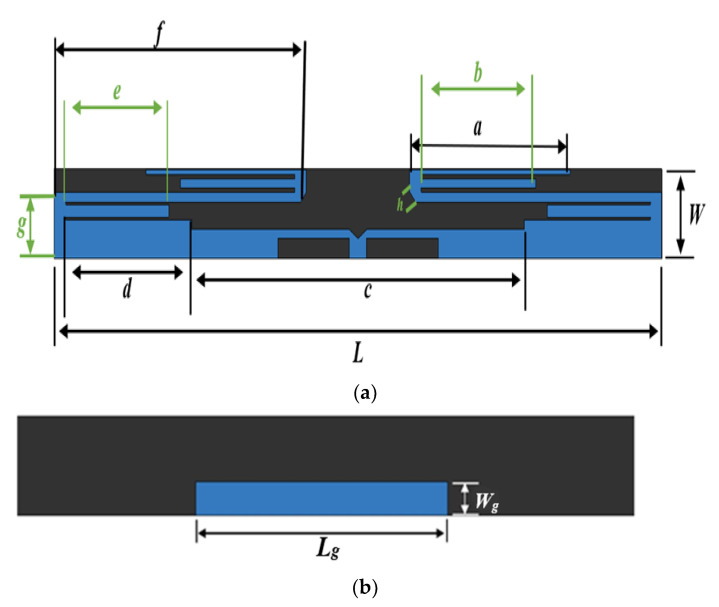
Geometry of the simulated monopole antenna design. (**a**) Front view and (**b**) back view.

Figure 1 depicts the geometry of the designed meander monopole antenna. The proposed antenna is made up of symmetric meandering elements. To meet the growing demand for communication equipment development, antenna research focuses on a few specific aspects, such as how to reduce antenna’s size while maintaining higher radiation efficiency. Meanwhile, as the size of small-scale integrated circuits becomes smaller, the size of communications equipment is reduced as well. When compared to other types of antennas, the meander line antenna provides the largest size reduction at a given frequency at the expense of a narrow bandwidth.

**Figure 2:**
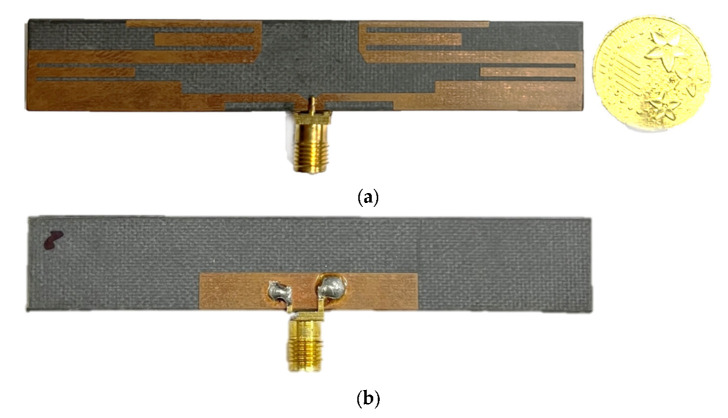
Prototype of realized monopole antenna array. (**a**) Front view and (**b**) back view.

The advantage of the meander line antenna is that it is relatively easy to catch a larger relative bandwidth. The fabricated antenna is fed using a 50 Ω SMA connector. The patch and ground plane are annealed by copper, and the substrate is Rogers RT5880 at 1.575 mm. The meander line monopole antenna prototype has been designed and constructed, and its photo can be seen in Figure 2.

All of the geometric parameters were considered and analyzed, and eventually, it was estimated that the following were the efficient parameters for the proposed scheme, as shown in Table 2. The designed antenna is smaller and much more compact, measuring only 90.86 × 15 mm^2^ at 2.4 GHz. Computer simulation technology (CST) was used to obtain the antenna’s simulation results.

## 3. Working Principles

Analyses and discussion of the simulated current distribution are conducted in order to gain an overview of the characteristics and behavioral standards of the proposed antenna design.

### Current Distribution

**Figure 3:**
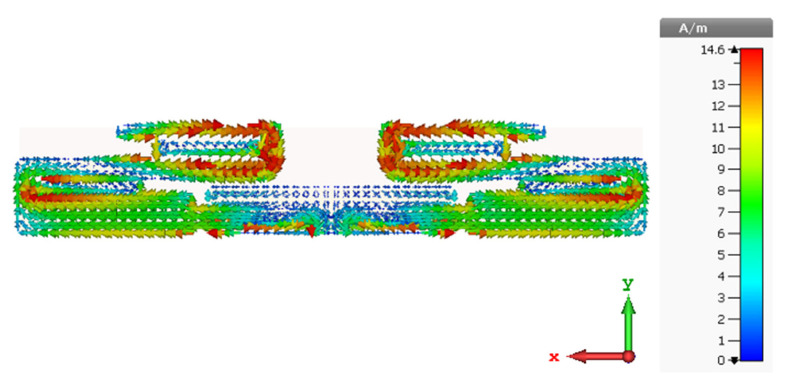
Depicts the proposed ML antenna’s surface current distribution.

Analysis and discussion of the simulated current distribution are conducted in order to gain insight into the properties and operating principles of the proposed antenna design. Figure 3 depicts the duplicated distribution of surface current for the proposed antenna at 2.4 GHz in order to save space and to be more concise. As can be seen from Figure 3, the current of the proposed printed ML monopole antenna is concentrated at the lower edge of the upper meander line and it can also be observed that the current concentrates on both sides of the antenna port, as illustrated in the Figure, which explains why the meander line increases the electrical line of the current path.

## 4. Parametric Study

The subsequent sections illustrate, for the purpose of improving comprehension, how the structure’s primary parameters have such an effect.

### 4.1. Upper Meander Line Length

**Figure 4:**
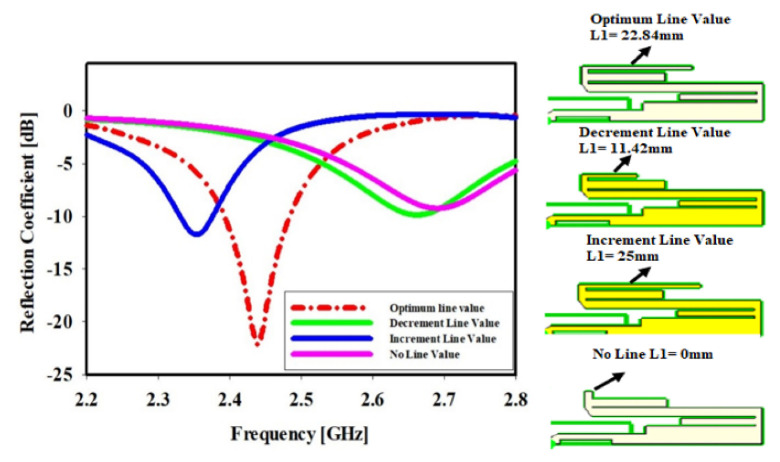
Upper meander line length effect, the sketches on the most−right side in Figure 4 show the geometries of the meander line changes.

Figure 4 depicts the effect of the upper meander line (ML) length and shows that changing the length will provide different reflection coefficients and slightly shift the frequency. It is clearly demonstrated that when the meander line is at its optimal length, L1olv = 22.84 mm, the reflection coefficient is −22.69 dB at the center frequency of 2.43 GHz, while halving the length of the meander line to L1dlv = 11.42 mm results in a reflection coefficient of −9.87 dB at the center frequency of 2.65 GHz. At 2.35 GHz, when the length of the ML is increased to L1ilv = 25 mm, the reflection coefficient decreases to −11.73 dB. Finally, when ML is removed, the reflection coefficient is −9.25 dB at the center frequency of 2.69 GHz with L1_no line_ = 0 mm.

We observed that there were no bands when we halved the length of the ML and removed it entirely. Nonetheless, as the ML length was increased, we observed a band with a slightly shifted frequency, whereas at the optimal line length, we observed a frequency concentration at 2.43 GHz. We are attempting to reduce or increase the length of the meander line in order to determine the effects of the different meander line lengths until we obtain the concentrate operation frequency. The figure below shows the geometric of the upper ML length.

### 4.2. Middle Meander Line Length

**Figure 5:**
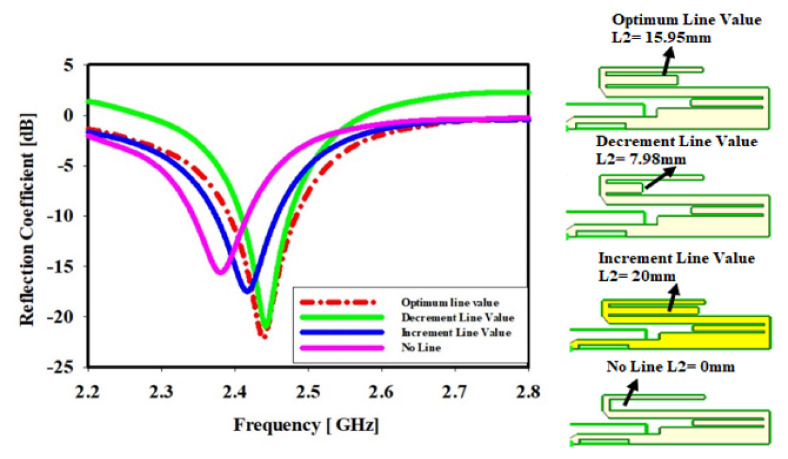
Middle meander line length effect: the sketches on the most-right side in Figure 5 show the geometries of the meander line changes.

Figure 5 indicates the impact of the middle meander line (ML) length, exhibiting the changing length outcomes in various reflection coefficients and a slight shift in frequency. It is clearly illustrated that when the meander line is at its optimal length, L1olv = 15.95 mm; the reflection coefficient is −22.16 dB at the center frequency of 2.43 GHz. 

On the other hand, the reflection coefficient is −23.72 dB at the center frequency of 2.44 GHz when the length of the meander line is cut in half and becomes L1dlv = 7.98 mm, while increasing the ML length to L1ilv = 20 mm results in a reflection coefficient of −17.48 dB at the center frequency of 2.41 GHz. Finally, after removing ML, L1_no line_ = 0 mm, the reflection coefficient is −15.62 dB at the 2.38 GHz center frequency. We can clearly see that the change in the middle ML length does not affect the bandwidth, but does affect the frequency range, as we can see in the figure below, where the frequency range has been shifted. The figure below shows the geometry of the middle ML length.

### 4.3. Lower Meander Line Length

**Figure 6:**
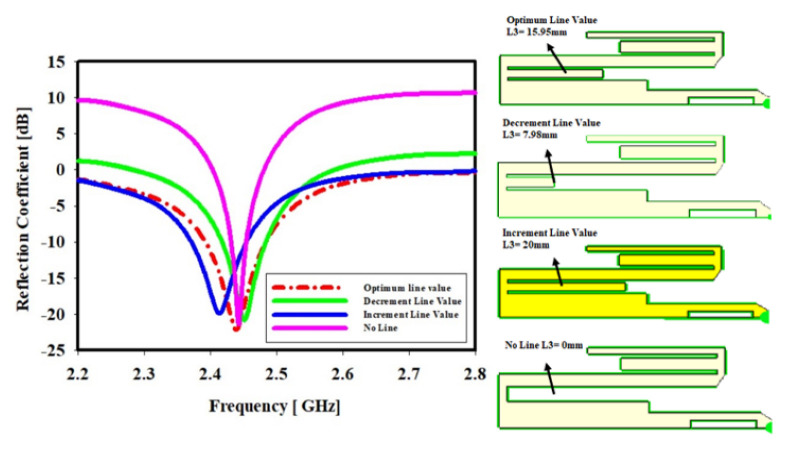
The effect of the middle−lower meander line length on the simulated reflection coefficient and the geometric of the lower ML length; the sketches on the most-right side in Figure 6 show the geometries of the meander line changes.

Figure 6 illustrates the effect of a lower meander line length whereas the frequency resonance has been slightly shifted. It is demonstrated unequivocally that when the meander line is at its optimal length, L1olv = 15.95 mm, the reflection coefficient is −22.16 dB at the 2.43 GHz centre frequency. While significantly reducing the meander line’s length to L1dlv = 7.98 mm, this results in a reflection coefficient of −23.44 dB at the 2.45 GHz centre frequency. When the ML length is maximised to L1ilv = 20 mm at 2.41 GHz, the reflection coefficient decreases to −19.85 dB. Finally, without the ML, the reflection coefficient is −32.37 dB at 2.44 GHz centre frequency with L1_no line_ = 0 mm.

As shown in the Figure below, the change in the lower ML length has no effect on the bandwidth but has an overall impact on the frequency range. The geometry of the lower ML length has been shown in Figure as well.

## 5. Results and Discussion

**Figure 7:**
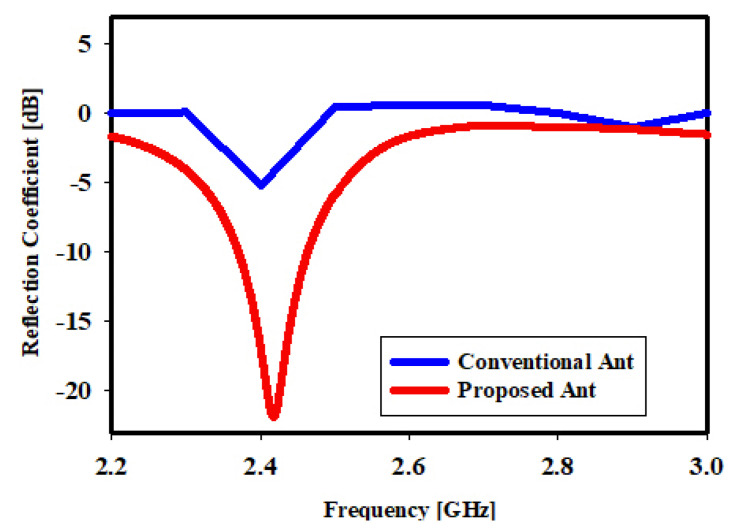
Performance comparison between the basic meander line antenna with the proposed design.

**Figure 8:**
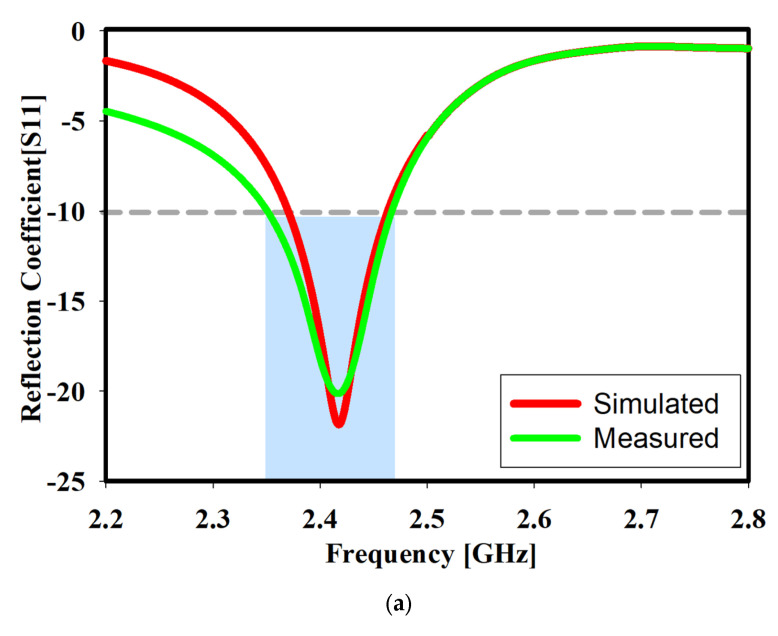

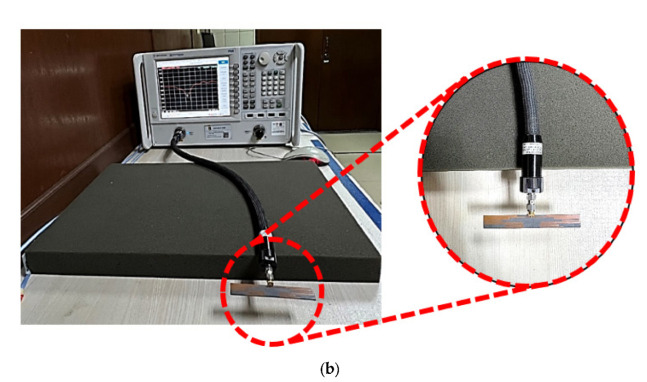
(**a**) The simulated and measured reflection coefficient of the suggested printed ML monopole antenna and (**b**) the reflection coefficient measurement setup.

Figure 7 shows a performance comparison between the conventional meander antenna in [32] and the proposed design, which has wider bandwidth. Figure 8a presents the meander line monopole antenna’s simulated and measured reflection coefficients. According to the Figure, the measured and simulated results are reasonably consistent with the perceived difference caused by material and hardware fabrication tolerances. The designed antenna’s bandwidth was discovered to be 94.2 MHz with a frequency range of 2.3685 GHz to 2.4643 GHz at less than −10 dB. Figure 8b presents the reflection coefficient measurement setup.

Prior to the selection and fabrication of a final structure, numerous essential parameters of the proposed antenna are analyzed to show better performance in terms of single-band operation, bandwidth, and compact size.

**Figure 9:**
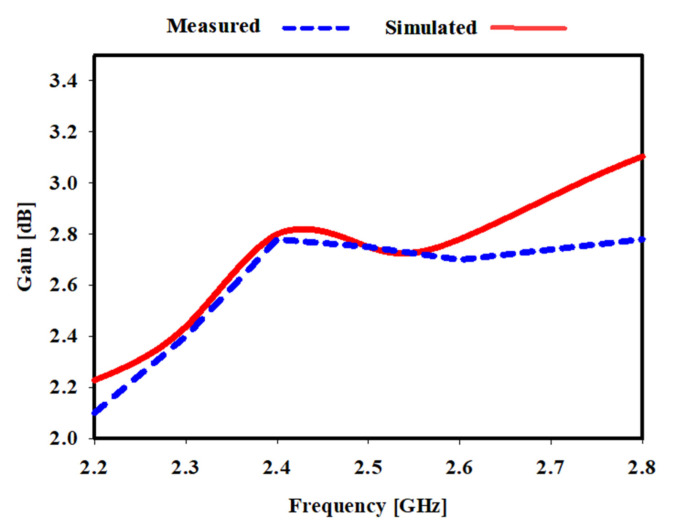
Simulated and measured gain for a monopole array antenna.

**Figure 10:**
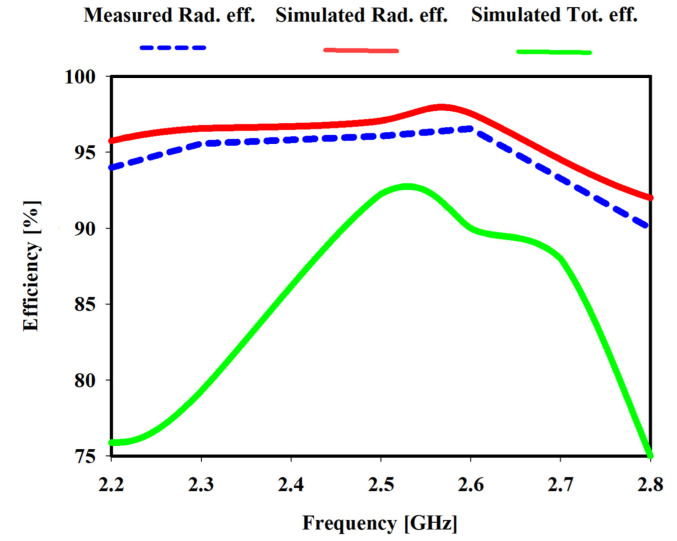
Simulated and measured efficiency of the proposed antenna.

Figure 9 illustrates the simulated and measured gain of the meander monopole antenna is 2.88 dB at 2.45 GHz. Furthermore, an agreement between the measured and simulated gains has been accomplished. Due to the meander line, the antenna gain with the meander line increased and was enhanced. Simulated and measured antenna efficiencies are shown in Figure 10. The proposed antenna has a maximum radiation efficiency of 99.4% at 2.4 GHz and a maximum total efficiency of 92% at 2.45 GHz.

**Figure 11:**
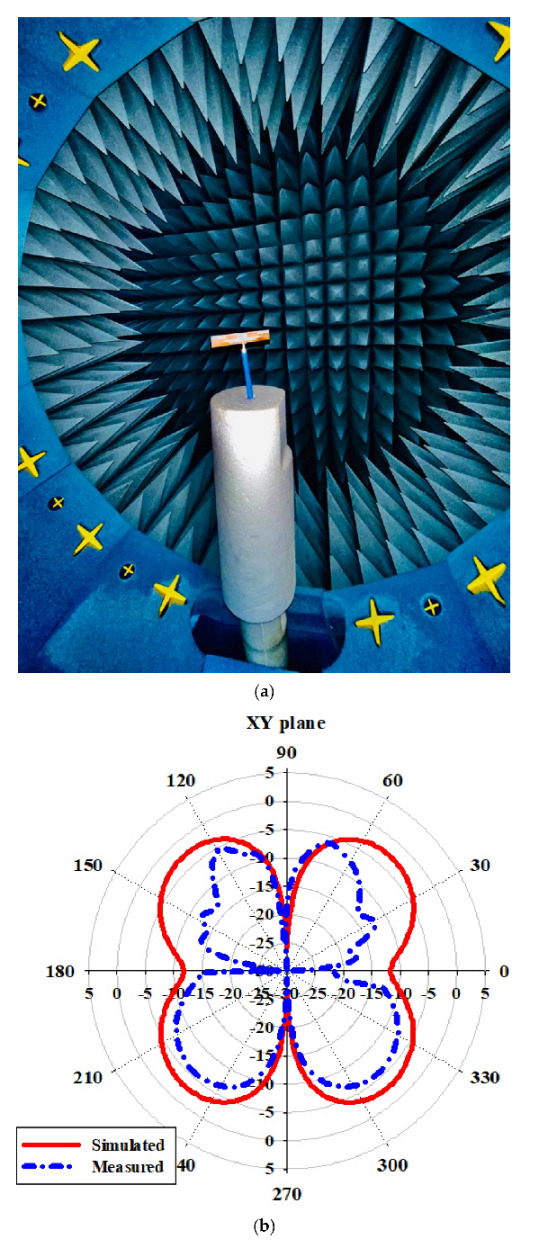

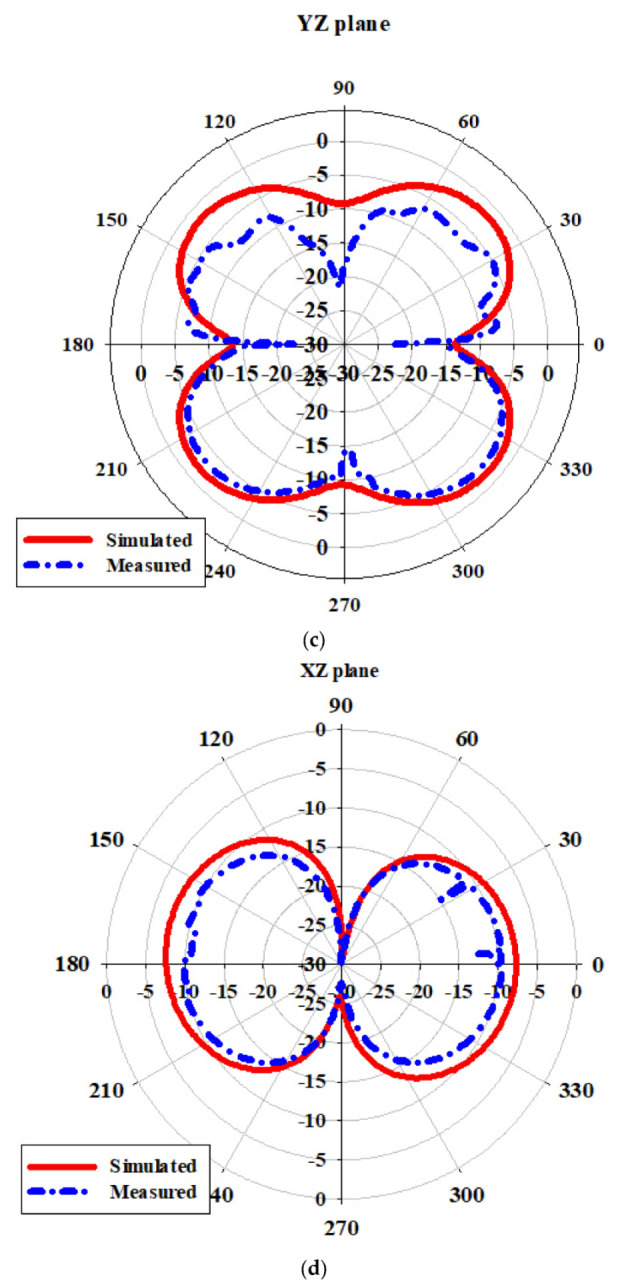
Radiation pattern, both simulated and measured, of the designed antenna operating at 2.4 GHz (**a**) radiation pattern measurement setup (**b**) xy, (**c**) yz, and (**d**) xz planes.

Figure 11 depicts the simulated, and measured radiation patterns in the proposed antenna’s E (φ = 0 deg) and H (φ = 90 deg) plans at 2.4 GHz. It indicates the radiated power’s variation in various spatial directions. We are able to see very clearly that the patterns are quasi-omnidirectional, and this makes them well suited for the reception of signals by WLAN wireless communication terminals.

## 6. Conclusions

In this article, the compact antenna array for 2.4 GHz WLAN has been demonstrated. It is shown that, by adjusting the length of the upper, middle, and lower patch, resonance can be adjusted widely within the frequency range prior to being optimized at 2.4 GHz frequency band. The proposed antenna array has achieved with 2.8 dBi gain and a bandwidth of 94.3 MHz with an efficiency level of 97%. The simulated and measured results are in concurrence. The proposed antenna has small dimensions in comparison to the earlier models, and the boundaries of performance were shown to be appropriate for WLAN programs in locations with poor coverage. Possible future modifications or improvements would be concentrated on enhancing the gain and other boundaries of performance. Upcoming work will be foreseen by using this antenna as a receiver in an energy harvesting system [2,33].

## Figures and Tables

**Table 2:** Optimised dimensions of the proposed monopole antenna.

Parameter	L	W	L_g_	W_g_	a	b	c	d	e	f	g	h
Dimension (mm)	90.86	15	33.3	6	22.8	15.9	41.3	24.8	15.9	37.5	10	2.5

## Data Availability

Not applicable.

## Abbreviations

olv	optimum line value
dlv	decrement line value
ilv	increment line value
